# Urethroplasty- a single centre single surgeon experience

**DOI:** 10.1007/s11845-024-03798-z

**Published:** 2024-09-03

**Authors:** Daniel Peter McNicholas, Alexander Taylor, Andrew D. Baird

**Affiliations:** Aintree University Hospital, Liverpool University Hospital Foundation Trust, Lower Lane, Fazakerley, Liverpool, England L9 7AL

**Keywords:** Anastomotic urethroplasty urethral stricture, Buccal mucosal graft, Urethral reconstruction, Urethroplasty

## Abstract

**Introduction:**

Male urethral stricture affects 100 in 100,000 men. These are investigated using uroflowmetry, retrograde urethrography and cystourethroscopy. Management is usually endoscopic with urethral dilation or direct visual internal urethrotomy, although they have high failure rates. It is now recommended that urethroplasty is performed earlier. In this study we have reviewed a single surgeons experience with urethroplasty and patient outcomes.

**Methods:**

We retrospectively reviewed a prospectively maintained database of all urethroplasty operations performed in our hospital over a 5 -year period.

**Results:**

Forty-five patients were identified, with a mean age of 46. The most common presenting symptom was poor flow (100%). Uroflowmetry was performed in 31 of 45 patients(69%). More patients had a urethrogram (58%) than flexible cystoscopy (38%). Most strictures were idiopathic (67%). Mean stricture length was 2.6 cm. 71% did not require any further intervention. Five patients required repeat surgery. Four required DVIU and one required a repeat urethroplasty.

**Discussion:**

The most popular techniques for urethroplasty in the UK are augmentation urethroplasty using a buccal mucosal graft and anastomotic urethroplasty, both of which we describe. There are variations in what is deemed as successful surgery. The most widely used definition is ‘the lack of need for any further operative intervention’. We have recently adopted Patient Reported Outcome Measures using a validated questionnaire to measure the patients perception of a successful outcome. Complex strictures have a higher incidence of complications. 42% of our cohort were complex and we describe results comparable to the published literature.

## Introduction

Male urethral stricture is defined by the European Association of Urology (EAU) as a narrowed segment of the anterior urethra due to a process of fibrosis and cicatrisation of the urethral mucosa and surrounding spongiosus tissue [1]. It is a disease that affects an estimated 10 in 100,000 in younger men and increasing as high as 100 in 100,000 in men over 65 in the United Kingdom [2]. It mostly affects the anterior urethra (92%). The bulbar urethra is affected in 46% of cases, 30% are in the penile urethra, the remainder are a mixture of both penile and bulbar urethra or pan-urethral [3]. The most common identifiable causes are iatrogenic injuries (38%) from catheterisation, hypospadias repair and transurethral surgery. Other causes include lichen sclerosis and trauma. However, a large proportion of cases are of unknown aetiology [3]. In the industrialised world, post- infection inflammation causes approximately 15% of urethral strictures [4]. The most common presenting symptoms are lower urinary tract symptoms (LUTS) such as poor flow, dribbling and incomplete emptying, and acute urinary retention, urinary tract infections, and difficult catheterisation [5]. Pain is a common feature amongst younger men. It can affect the urethra or the bladder, and it usually resolves with surgery [6]. Management of a urethral stricture depends on the stricture characteristics. To evaluate this, it is recommended by the EAU to perform uroflowmetry and post void bladder scan, although it is recognised that results are subjective and potentially unreliable, a uroflowmetry showing reduced maximum flow rate and prolonged plateau flow is characteristic of a urethral stricture [1, 7]. Retrograde urethrography is widely recognised as the investigation of choice and is recommended by the EAU [1]. It gives information about stricture length, location, the number of strictures and other pathology such as false passages [8]. Cystourethroscopy can be used to visually diagnose a urethral stricture however it cannot assess stricture length and may not detect multiple strictures [9]. There is weak evidence to support its use if imaging has already been performed [1]. Management of urethral strictures in the first instance is usually endoscopic with urethral dilation or direct visual internal urethrotomy (DVIU) [10]. In the United States, studies have shown that DVIU is favoured by Urologists [11, 12]. Urethral dilation has been shown to have similar surgical outcomes to DVIU [13]. It is well known that endoscopic stricture repair has high rates of failure, stricture recurrence has been documented between 50–90% [14, 15]. Furthermore, repeated endoscopic repairs can lead to more complex strictures, making definitive repair more difficult [16, 17]. It is now recommended that urethroplasty is performed earlier, and to avoid repeated endoscopic procedures [18]. There is evidence of superior outcomes from urethroplasty than endoscopic treatments [19, 20]. In this study we have reviewed a single surgeons experience with urethroplasty and patient outcomes.

## Methods

We retrospectively reviewed a prospectively maintained database of all urethroplasty operations performed in our hospital over a 5 -year period from January 1st 2015- December 31st 2019. All operations were performed by a single consultant urological surgeon.

All patient records were reviewed, and any duplicates were removed. All clinical details were anonymised and recorded. The patient data was analysed by two separate people (DM and AT) to ensure accuracy of the results.

Data collected includes patient demographics, pre and post- operative uroflows, pre-operative flexible cystoscopy and urethrography, past medical history, previous surgical procedures, post-operative follow up and complications.

## Results

In total 45 patients were identified, with a mean age of 46 (22–67) (Table 1).

**Table 1:** Socio-demographic and clinical characteristics of the study group

Age in years (range)	46 (22–67)
Aetiology; N (%)	
Idiopathic	31 (67%)
Congenital	7 (13%)
Trauma	5 (11%)
Iatrogenic	4 (9%)
Type of operation; N (%)	
End-to-end	11 (24%)
Buccal mucosa graft (BMG)	30 (67%)
2 stage substitution with BMG	4 (9%)
Location of stricture; N (%)	
Bulbar	30 (64%)
Bulbo-prostatic	6 (14%)
Penile	5 (11%)
Peno-bulbar	5 (11%)

### LUTS and investigations

The most common presenting symptom was poor flow (100%). Other symptoms include acute urine retention (13%), haematuria (13%) and UTI (6.7%). Uroflowmetry was performed in 31 of 45 patients (69%). The mean maximum flow rate (Q-Max) was 8.4 ml/second (range: 1-20 ml/second). More patients had a urethrogram performed (58%) compared to flexible cystoscopy (38%), 2 patients didn’t have either procedure documented.

### Previous surgeries

Thirty-three patients (73%) had previous DVIU, 22% had urethral dilation, 20% performed ISC and 15% had a supra-pubic catheter (SPC) in-situ. Previous hypospadias repair Had been performed in 13% of patients, 6.7% had had a previous TURP and 4.4% had undergone prior radical prostatectomy.

### Stricture data

Most strictures were idiopathic in origin (67%), congenital strictures accounted for 13%, there were 11% traumatic and 9% of iatrogenic aetiology (Table 1). Stricture location was as follows; bulbar urethra (64.4%); bulbo-prostatic (14%); peno-bulbar and penile strictures 11.1% each (Table 1). In 35 patients the length of their stricture was formally measured. Mean stricture length was 2.6 cm (0.4 cm-9 cm). Median stricture length was 2.5 cm. Twenty-five patients had strictures > 2 cm.

### Procedure

Surgical time ranged from 130 to 424 min, with a mean surgical time of 211 min. The median surgical time was 192 min. The most common operation was augmentation urethroplasty with buccal mucosal graft (BMG), accounting for 34 operations (76%). Four of these were two stage substitution urethroplasties. Anastomotic urethroplasty was performed in 11 patients (24%) (Table 1). Inpatient length of stay ranged from 1 to 5 days (mean 1.8 days, median 2 days). All patients had a trial without catheter 2 weeks post operatively and were followed up by the consultant initially at 3 months.

### Post- operatively

There were 16 Clavien- Dindo (CD) Grade-1 complications and five CD Grade-2 complications. There were no CD Grade 3 or 4 complications (Table 2). The CD grade-1 complications were all pain management related.

**Table 2:** Clavien-Dindo Classification and associated complications

Clavien-Dindo Classification	Number of Patients
Grade 1	16
Grade 2	5
	- Scrotal haematoma
	- Post operative anaemia
	- Wound infection
	- Urosepsis with urine retention
	- Urosepsis with pelvic collection
Grade 3	0
Grade 4	0

The CD Grade-2 complications consisted of post operative anaemia, scrotal haematoma, wound infection, urosepsis with urine retention and urosepsis with a pelvic collection. All cases were managed with conservative medical management (Table 2).

### Outcomes

Of the 45 patients that had urethroplasty performed, five (11%) required repeat surgery for their strictures. Four patients (8%) required urethral dilation only, but no surgical intervention. Seven patients (15%) were required to perform long term ISD post urethroplasty. In total, 32 patients (71%) did not require any surgery or further intervention, such as urethral dilatation or ISD, post- operatively (Table 3).

**Table 3:** Outcome in terms of subsequent interventions for patients that have had urethroplasty

Post Urethroplasty Interventions	Number of Patients
No intervention required	32 (71%)
Urethral dilation only	4 (8%)
Intermittent Self Dilation	7 (15%)
Surgery	5 (11%)

Five patient required repeat surgery. Four required DVIU and one required a repeat urethroplasty. Two of the DVIU patients and the urethroplasty patient all initially had urethral dilation prior to their surgical intervention. Of the patients requiring DVIU none had any acute post- operative complications. One other patient required surgery after his urethroplasty, he developed intractable LUTS due to bladder overactivity and was managed with intra-vesical botulinum toxin injections and artificial urinary sphincter.

Post operative uroflows were performed in 26 patients. The mean Q-max was 22.3 ml/s and the median was 19 ml/s. A total of 19 patients completed both pre and post- operative uroflows. There was an improved flow in 17 patients (89%). The mean improvement was 16.1 ml/s (± 12.7 ml/s). The median improvement was 9 ml/s. Eleven patients had < 10 ml/s improvement in uroflow, of these 7 required further intervention.

In total, seven patients had urethral dilatation performed post urethroplasty surgery. One of these patients had a further DVIU and urethroplasty, one had further DVIU and performs ISD, and another patient performs regular ISD. Four patients required no further intervention or ISD (Table 4). Five of these seven patients were considered complex and are discussed further below.

**Table 4:** Breakdown of total subsequent interventions and surgery for patients that have had urethroplasty

Interventions and surgery	Number of Patients
Urethral dilation only	4
DVIU only	3
Urethral dilation, subsequent DVIU, then urethroplasty	1
Urethral dilation and subsequent DVIU	2

Seven patients are required to perform long term ISD after their urethroplasty. Two of these patients had DVIU and one had urethral dilatation.

Five patients had hypospadias repair in childhood. They all underwent augmentation urethroplasty with BMG. Three of these were two stage substitution urethroplasties. One patient who underwent two stage substitution urethroplasty subsequently required urethral dilatation followed by a repeat urethroplasty with BMG. One other patient who had a single stage procedure subsequently required urethral dilation, DVIU and long term ISD.

## Discussion

Urethroplasty is strongly recommended as the definitive treatment for recurrent urethral stricture by the EAU guidelines 2023 [1]. The most popular techniques for urethroplasty in the UK are augmentation urethroplasty using a buccal mucosal graft which was first described by Humby in 1941, and anastomotic urethroplasty [(https://www.urologynews.uk.com/media/9093/uroma18-urethroplasty-v2.pdf), 21]. The type of urethroplasty operation is dependent on the stricture characteristics. Short strictures are amenable to anastomotic urethroplasty, however longer or more complex strictures have better outcomes from an augmentation urethroplasty [22, 23]. In our study, augmentation urethroplasty accounted for 76% of our procedures and anastomotic urethroplasty accounted for 24% of them.

In our study, for patients who had both pre- and post- operative uroflow performed there was a mean improvement in urine flow of 16 ml/s. Erickson’s paper made an interesting observation that change in flow rate post urethral reconstruction may be related to risk of recurrence. They identified that a change of flow less than 10 ml/s had a 92% sensitivity and 78% specificity for those at risk of stricture recurrence [24]. It has also been shown that patients voiding symptoms should be considered as well as looking at the uroflow curves when diagnosing stricture recurrence [25]. On review of our patient records, over 70% of patients required no further urological intervention (eg. dilation, ISD, urethroplasty) for their urinary strictures. 90% of patients didn’t require any further surgical intervention. Of the repeat surgeries performed, 80% were endoscopic, while one patient required repeat urethroplasty. It is not uncommon for patients to require local anaesthetic urethral dilation, or to perform intermittent self-dilatation (ISD) post urethroplasty. In our patient cohort 15% of patients required ISD and 8% required a single urethral dilation with no other interventions. Stricture recurrence has been defined as requirement for further surgery [26]. In our study, 10% of patients required further surgery. There are varying success rates reported in the literature for urethroplasty procedures, depending on the type of procedure performed. Barbagli’s retrospective review of 375 patients undergoing one of 3 urethroplasty techniques, had an overall success rate of 83.5%. The success rate of the various techniques differed greatly. Primary anastomosis had 91% success, onlay grafting techniques had 82% success and augmented anastomotic repair using penile skin grafts had 60% success. Recurrence rates in the literature vary from 10–40% [25–27].

It is worth pointing out that in our study, the ‘success’ of our urethroplasty surgeries has been measured using the most widely used definition- the lack of need for any further operative intervention. We also used uroflow as a barometer of success, but since less than half of our patients completed both pre and post operative uroflows, we can see that its’ use is limited. There are well known reasons why it can be challenging to consistently get a uroflow for every patient in clinic, thus representing a limitation of this method of measurement of success. Furthermore, it has been shown that uroflow parameters failed to demonstrate significant contribution to patient satisfaction [28]. A method to measure success which we have recently adopted is Patient Reported Outcome Measures (PROM) using a validated questionnaire, such as the Urethral Stricture Surgery PROM (USS-PROM), to assess patient satisfaction before and after their operation. The USS PROM was specifically developed in 2011 by Jackson et al. for patients undergoing urethral stricture surgery [29]. It examines details such as LUTS, quality of life, overall satisfaction and overall health. Interestingly, using PROM’s, Kessler has shown that only 78% of patients with clinical success are actually satisfied [28], meanwhile 80% of those with clinical failure are satisfied with their outcome [30]. So, it is clear that PROM’s have a key role to play in assessing the outcome of urethroplasty.

Post- operative complications are an inevitable part of urethroplasty, particularly complex operations with prolonged operative times. In our cohort of patients 35% developed Clavien-Dindo (CD) Grade 1 complications and 11% had CD Grade 2 complications. This compares favourably with other studies such as Hussein et al. who had 36% CD Grade 1 complications [31]. Another study by Spilotros et al. describes complications such as graft contracture, graft failure, fistula, mouth bleeding, wound infection and perineal haematoma. They identified a complication rate of around 12% and a re-stricture rate of 20% [32]. Spilotros et al. also demonstrated that longer stricture length increases the risk of failure, with 7% risk of failure for strictures < 4 cm, while it is 20% for those 4-8 cm in length and 30% for those > 8 cm. It is worth noting that we found good outcomes from patients with stricture lengths > 4 cm, with only one patient requiring further urethral dilation. A few studies described strictures as complex when patients had a history of previous hypospadias, BXO or recurrent stricture disease. All these factors were associated with a higher risk of stricture recurrence post urethroplasty [33, 34]. In our patient group, 42% were considered complex cases with BXO, previous hypospadias repair, strictures > 4 cm as well as other factors such as previous prostatectomy and urethral trauma. Of these patients, 26% required further intervention in the form of urethral dilatation, DVIU or further urethroplasty. One patient had suffered a burns injury and had a stricture > 4 cm long which was managed with single stage urethroplasty with BMG. He required one dilatation of his urethra in the first year post- operatively. Another patient had a traumatic urethral dissection injury. He required a two-stage substitution urethroplasty. He required one urethral dilatation one year after his second stage procedure. One other patient who had previous radical prostatectomy required urethral dilatation > 1 year post- urethroplasty with BMG. As was described in our results, 5 patients had a previous hypospadias repair as a child. One of these required further urethral dilatation followed by a repeat urethroplasty with BMG, while a second required urethral dilation, DVIU and long term ISD.

In summary, we have reviewed our patient cohort with regards to presentation, investigations, surgeries along with the follow up and outcomes. We have shown good outcomes for patients who have urethroplasty procedures in our centre. Our highly experienced surgeon has favourable outcomes which are comparable to the published literature. Owing to patient factors and tertiary referrals, the patient cohort is complex. The outcomes of this study are very encouraging and will add to the literature in this field. Further larger studies do need to be carried out to identify prognostic indicators for good outcomes to help improve the outcomes for our patients. We advocate the use of PROM’s to enhance the follow up of patients and get a better global picture of what represents surgical ‘success’ for the patient.

## Data Availability

The data that support the findings of this study are available from the corresponding author, [DM], upon reasonable request.
